# Patient experience of and barriers to the eye examination

**DOI:** 10.1038/s41433-026-04499-w

**Published:** 2026-05-06

**Authors:** Siyuan Jabelle Lu, Shenouda Girgis, Peter Shah, Graham A. Lee

**Affiliations:** 1 Brisbane North Eye Centre, Brisbane, QLD Australia; 2 University of Sydney, Sydney, NSW Australia; 3 Greenlane Eye Clinic, Te Whatu Ora Te Toka Tumai Auckland, Auckland, New Zealand; 4 Birmingham Institute for Glaucoma Research, Institute of Translational Medicine, University Hospitals Birmingham NHS Foundation Trust, Birmingham, UK; 5 Centre for Health & Social Care Improvement, University of Wolverhampton, Wolverhampton, UK; 6 Queensland Academy of Ophthalmology, University of Queensland, Brisbane, QLD Australia

**Keywords:** Health services, Public health

## Abstract

**Background:**

Ophthalmic examination is central to the diagnosis and monitoring of eye disease. However, there is a paucity of qualitative studies about the patient’s experience. The Patient Experience of Eye Examination eValuation Study (PEEEVS) was designed to evaluate patient experiences of key examination-related components of routine ophthalmic care.

**Methods:**

PEEEVS employs a cross-sectional, mixed methods design combining quantitative visual analogue scales (VAS) with qualitative semi-structured interviews. Data from 203 patients (M:F - 101:102) were analysed with respect to their experiences of topical anaesthesia, optical coherence tomography (OCT) imaging, tonometry, slit lamp examination, and mydriasis.

**Results:**

Quantitative analysis indicated predominantly favourable responses, with median VAS scores ranging between 88 and 89 for all tests. In contrast, qualitative findings identified specific areas of concern, including challenges with maintaining proper positioning during slit lamp examination and OCT, particularly for individuals with pre-existing neck/back pain, larger body habitus, or advanced pregnancy.

**Conclusions:**

While participant experience of most monitoring tests was good, specific examination aspects can impact patient comfort and potentially affect long-term adherence to ocular monitoring. The study highlights the need for targeted considerations, such as ergonomic improvements and enhanced patient communication strategies to alleviate discomfort and support sustained engagement in ophthalmic care.

## Introduction

Chronic eye disease requires lifelong monitoring with a series of tests to detect progression and optimise therapeutic interventions. These tests involve the use of topical anaesthetic drops, visual field testing, optical coherence tomography (OCT), tonometry, examination with a slit lamp, and mydriasis. While these procedures are routine for clinicians, they may present unique physical or psychological challenges to patients, potentially influencing adherence to monitoring and treatment. There is growing recognition of the barriers associated with visual field testing [1, 2]. However, patient perspectives on other components of the ophthalmic examination have not been widely studied in the peer-reviewed literature.

The Patient Experience of Eye Examination eValuation Study (PEEEVS) was designed to address this gap by employing a mixed methods approach to evaluate patient-reported experiences, attitudes, and barriers during routine ophthalmic examinations in Australian private practice settings [1]. The findings from Lu et al. on visual field testing highlighted challenges such as test-related anxiety and fatigue, physical discomfort, and patient frustration and scepticism regarding the test’s clinical utility [1]. It also found that patients with glaucoma perceived visual field testing more negatively than patients with other ocular diagnoses. These findings led to key recommendations focused on practical considerations, such as allowing patient-initiated breaks and ensuring patients understand the inherently challenging nature of the test [1].

The aim of this study is to provide a quantitative and qualitative evaluation of the patient experience of common components of the ophthalmic examination [1]: topical anaesthesia [2], OCT imaging [3], tonometry [4], slit lamp examination and [5] mydriasis. By identifying patient-reported experiences throughout these procedures, the goal is to refine communication and guide technical adjustments, ultimately improving patient experiences along their eye care journey.

## Methods

PEEEVS is a mixed methods study designed to evaluate patient experiences across common ophthalmic diagnostic procedures in an ophthalmic practice setting. The design and implementation of PEEEVS have been described in detail [1].

### Design and data collection

**Fig. 1:**
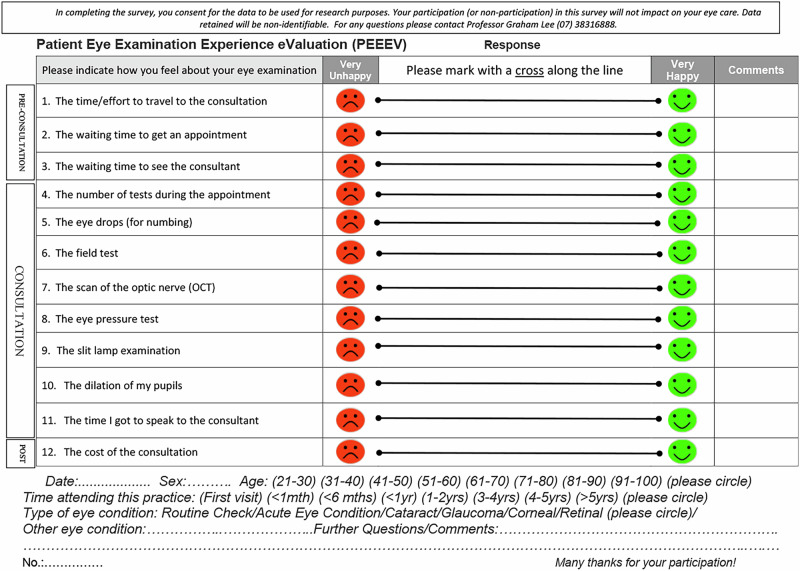
The Patient Experience of Eye Examination eValuation Study (PEEEVS) instrument.

PEEEVS is a 12-item instrument combining visual analogue scales (VAS) with semi-structured interviews Fig. 1. Patients rated their experiences of each item on a 100 mm VAS (0 = “very unhappy,” 100 = “very happy”). While the specific combination of examination-related items assessed in this study is novel, the 100 mm VAS-based survey format has been previously implemented within an Australian cohort of glaucoma patients in a private outpatient setting and demonstrated feasibility and interpretability for assessing patient-reported experience in this population [3]. Open-ended prompts were used to collect qualitative data, with contemporaneous verbatim notes cross-verified post-interview for accuracy. The study followed a concurrent nested design, where the qualitative data was intended to be supplementary to the quantitative data [4]. Therefore, comments such as “I am happy with it” were excluded from qualitative analysis as they did not provide further value to the VAS scores. The instrument was piloted in 20 patients prior to study commencement to assess clarity, comprehensibility and participant engagement, during which no issues were identified and no modifications were required.

Patients received oxybuprocaine hydrochloride 0.4% for topical anaesthesia. OCT was conducted using either the Cirrus HD-OCT (Carl Zeiss, Dublin, California, USA) or Nidek OCT (Nidek Co. Ltd., Gamagori, Japan). Test duration for each eye was approximately three minutes, contingent upon the number of attempts required to obtain images of satisfactory quality. Intraocular pressure measurements were taken with rebound tonometry (iCare Finland Oy, Vantaa, Finland) and/or Goldmann applanation tonometry (GAT) on the slit lamp. For mydriasis, patients received tropicamide 1% and/or phenylephrine hydrochloride 2.5%.

### Participant selection

This study was conducted at City Eye Centre and Brisbane North Eye Centre in Brisbane, Australia, between June and August 2022. Ethical approval was obtained from the University of Queensland Human Research Ethics Committee (Project number 2022/HE000450), and the study adhered to the principles outlined in the Declaration of Helsinki. All patients received detailed information sheets and provided written informed consent.

Eligible patients were adults aged ≥21 years presenting for consultation. Exclusion criteria included inability to provide consent, age <21 years, language or comprehension barriers, advanced vision loss preventing VAS completion, acute ocular pathology, or recent ophthalmic surgery ( < 3 months). Eligible patients were consecutively approached post-consultation and invited to participate. Given the exploratory and descriptive nature of this mixed-methods study, no formal a priori power calculation was performed. Instead, the target sample size was guided by pragmatic considerations informed by related studies in the literature and qualitative methodology principles [2, 4, 5]. A sample size of approximately 200 participants was considered sufficient to detect meaningful differences between key demographic subgroups in non-parametric analyses and to achieve thematic saturation for qualitative data.

### Data analysis

Quantitative data were analysed using Stata/IC 16.1 (StataCorp, Texas, USA). Descriptive statistics, including mean ± standard deviation (SD), median, and interquartile range (IQR), were calculated for VAS scores. Non-parametric comparisons were performed using the Mann-Whitney U test for self-identified sex (male vs. female) and primary eye condition (glaucoma vs. other ocular pathologies). The Kruskal-Wallis test was applied to compare VAS scores across age groups (21–30, 31–40, 41–50, 51–60, 61–70, 71–80, 81–90, 91–100 years), with significant results further analysed using Dunn’s pairwise comparison.

Qualitative data were thematically analysed using a general inductive approach, situated within a realist epistemological stance [6]. This involved iterative close reading and coding of interview data to derive themes from participant responses without imposing a pre-existing theoretical framework, with the assumption that participants’ accounts reflect their lived experiences of ophthalmic examinations. Dual independent coding (SJL and GAL) was performed using NVivo 12, with themes refined and agreed upon through team consensus, consistent with established guidance for inductive qualitative analysis and reporting standards [6, 7].

## Results

### Quantitative

Of 218 patients approached during the study period, 203 (M:F – 101:102) consented to participate. The remaining patients declined participation or did not meet eligibility criteria. As shown in Table 1, the age group with the highest proportion of patients were 71-80 years of age (*n* = 70, 34.5%). The majority of patients (*n* = 146, 71.9%) were of the age group 61–70 and older. Patients had a range of ocular conditions, with glaucoma being the most common (*n* = 115, 56.7%).

**Table 1:** Patient and disease characteristics (*n* = 203).

Variable	*n* (%)
Age group (years)	
21-30	8 (3.9%)
31–40	5 (2.5%)
41–50	13 (6.4%)
51–60	31 (15.3%)
61–70	47 (23.2%)
71–80	70 (34.5%)
81–90	27 (13.3%)
91–100	2 (1.0%)
Sex	
Male	101 (49.8%)
Female	102 (50.2%)
Primary ophthalmic condition	
Glaucoma	115 (56.7%)
Corneal	42 (20.7%)
Cataract	17 (8.4%)
Retinal	14 (6.9%)
Other ocular pathology	15 (7.4%)

The VAS score responses for each of the consultation elements are shown in Table 2. Responses to all 5 questions were skewed towards the ‘very happy’ end of the VAS, with both mean and median VAS scores above 80. This indicates that these tests are generally well accepted, although mydriasis appears to be the least favoured examination component.

**Table 2:** VAS score responses for consultation elements (*n* = 203).

	Mean (SD)	Median; IQR	Skew
Topical anaesthesia	83.46 (18.17)	89; 80-93	-2.35
OCT	84.18 (13.87)	88; 79-93	-1.41
Tonometry	84.91 (15.14)	89; 80-93	-2.21
Slit lamp examination	84.38 (15.46)	89; 79-93	-1.78
Mydriasis	80.72 (21.08)	88; 78-93	-1.87

The analysis of VAS score responses against demographic variables is shown in Table 3. Most groups showed favourable scores with no statistically significant differences. However, there were significant differences in VAS scores between various age groups for tonometry (*p* = 0.044) (See supplementary information for Dunn’s pairwise comparison of VAS scores between different age groups), where older age groups indicated higher VAS scores for tonometry compared to younger age groups, particularly in the range of 21–30 years.

**Table 3:** VAS score responses according to participant demographics (*n* = 203).

	Topical anaesthesia (*n* = 183; mean [SD])	*p* value	OCT (*n* = 187; mean [SD])	*p* value	Tonometry (*n* = 199; mean [SD])	*p* value	Slit lamp examination (*n* = 201; mean [SD])	*p* value	Mydriasis (*n* = 175; mean [SD])	*p* value
Age group (years)										
21-30	80.2 (8.9)		74.4 (12.0)		66.9 (27.9)		78.8 (20.2)		75.0 (20.5)	
31-40	95.0 (6.0)		96.6 (8.4)		93.0 (6.1)		94.2 (5.9)		96.2 (4.1)	
41-50	81.0 (13.7)		77.0 (20.8)		76.5 (18.8)		82.2 (13.5)		76.4 (23.4)	
51-60	74.1 (30.4)		84.0 (14.7)		82.7 (16.6)		80.2 (21.2)		74.0 (27.1)	
61-70	83.4 (20.0)		85.6 (13.6)		86.4 (15.9)		85.6 (16.0)		83.4 (22.0)	
71-80	86.2 (12.1)		84.1 (13.1)		86.5 (11.7)		84.7 (12.6)		80.0 (19.8)	
81-90	86.2 (10.5)		85.7 (10.9)		88.0 (9.7)		86.8 (10.8)		84.5 (14.3)	
91-100	91.5 (12.0)		88.0 (17.0)		94.0 (80.5)		90.0 (14.1)		90.0 (14.1)	
		0.25		0.23		0.04*†		0.47		0.83
Sex										
Male	81.9 (19.6)		84.8 (13.4)		86.4 (13.0)		85.2 (15.0)		82.2 (19.7)	
Female	85.1 (16.5)		83.5 (14.5)		83.4 (17.0)		83.6 (15.4)		79.0 (22.5)	
		0.15		0.57		0.34		0.38		0.51
Primary eye condition										
Glaucoma	84.2 (18.3)		84.4 (13.6)		87.0 (12.0)		85.9 (13.5)		81.2 (20.7)	
Non-glaucoma	82.4 (18.1)		83.9 (14.4)		82.1 (18.2)		82.4 (16.9)		80.1 (21.7)	
		0.36		0.84		0.16		0.32		0.94

^*^Statistical significance; *p* < 0.05.

† See Appendix for Dunn’s pairwise comparison.

With the exception of topical anaesthesia, males appeared to indicate more positive experiences across all the examinations, but this did not achieve statistical significance. There were also no statistically significant associations found between patients with glaucoma and patients without glaucoma. However, VAS scores were generally higher in the former group.

### Qualitative

Table 4 shows the number of qualitative responses for each question. The selection of excerpts was guided by its ability to exemplify the central themes that emerged [8]. Each quotation is accompanied by the participant’s study number, age group, and sex.

**Table 4:** Number of qualitative responses per PEEEVS item (*n* = 203).

Question	*n* (%)
Topical anaesthesia	23 (11.3)
OCT	9 (4.4)
Tonometry	19 (9.4)
Slit lamp examination	25 (12.3)
Mydriasis	22 (10.8)

### Topical anaesthesia

20 (9.8%) patients described a stinging sensation upon the instillation of topical anaesthetic. These patients, however, tend to express an understanding of its necessity to facilitate subsequent parts of the examination, and some described heightened discomfort when their eyes are inflamed.

It stings but it makes the rest of the appointment ten times easier. (P52, 21–30, male)

Only when my eye is inflamed, it really stings. (P17, 21–30, male)

### OCT

A small number of patients (*n* = 6, 3.9%) described physical discomfort, usually due to positioning, when undergoing OCT.

It’s hard to get comfy, you feel a kink in your neck. (P6, 71-80, male)

### Tonometry

Most patients who provided comments on tonometry differentiated between GAT (*n* = 9, 4.4%) and rebound tonometry (*n* = 6, 2.9%), and some of these patients expressed preference for the latter. The contact of the GAT probe with the eye was described as slightly uncomfortable. In contrast, rebound tonometry caused feelings of apprehension due to the flickering motion of the probe. Some also noted that the nature of the discomfort was more psychological than physical.

It is a bit uncomfortable but only because it touches your eye. (P180, 71–80, male)

I just want to jump but it is not painful or anything. (P65, 71–80, female)

The flickering can get to me. (P29, 51–60, female)

It is just the idea of someone putting something in my eye. (P57, 41–50, female)

It is much better nowadays with the handheld device. (P54, 71–80, male).

### Slit lamp examination

23 (11.3%) patients described physical discomfort with the slit lamp examination. This is broken down into two subthemes. The first is the exacerbation of pain from pre-existing neck and back issues, often due to the prolonged and specific positioning required.

I have a bad neck. The way my forehead and chin have to go kinks my neck so it can get quite uncomfortable. (P190, 71–80, male)

I have a spine problem, and it becomes quite uncomfortable when you have to sit in a particular way and lean forward for so long. (P80, 71–80, female)

Another subtheme was discomfort and perceived awkwardness for individuals with a larger body habitus. Some of these patients appear to internalise these challenges as personal issues, attributing the discomfort to their own physical characteristics rather than to the slit lamp design.

I have to stand for the slit lamp because my body size is so large. (P163, 71–80, female)

Now I am fat, so I find it squeezing my tummy very hard, which is uncomfortable. But that is my own problem. (P155, 61-–70, female)

I am 7 months pregnant, so I can’t sit properly and have to lean forward quite awkwardly. (P168, 21–30, female).

### Mydriasis

11 (5.4%) patients described mydriasis as causing significant, albeit temporary, disruptions to their normal functioning. These comments mainly point to feelings of disorientation, dizziness, and heightened sensitivity to light, persisting for several hours post-procedure. Some describe a loss of spatial awareness and encountered challenges in navigating their surroundings during this time.

It’s a pain. You don’t know where you are walking for a few hours, but you learn to put up with it. (P19, 71–80, male)

You feel a little bit drunk for a few hours. (P39, 71–80, female)

I feel a bit knocked out for a couple of hours (P148, 71–80, female)

Dizziness and brightness that lasts hours (P168, 21–30, female)

Stinging on application was also mentioned by 6 (2.9%) patients, to various degrees of tolerability.

The drops sting but I just accept it as a part of the process. (P73, 81–90, female)

They do sting a lot. (P97, 61–70, female).

## Discussion

While the clinical utility of ophthalmic tests remains the foremost consideration in the diagnosis and monitoring of eye disease, patient experience plays a pivotal role in shaping long-term treatment attendance, and hence, disease outcomes [9]. By identifying common sources of discomfort or dissatisfaction, whether physical or psychological, clinicians can implement practical measures to improve the testing experience. Furthermore, insights from patient feedback are necessary to help guide more patient-friendly equipment designs.

This paper follows prior findings by Lu et al. [1] regarding visual field testing experiences, using the same methodology, and presents findings on other key components of the ophthalmic examination, namely topical anaesthesia, OCT, tonometry, slit lamp examination, and mydriasis. To our knowledge, patient experiences of many of these components have not been measured or explored previously in the peer-reviewed literature.

Patients rated all the tests favourably, with median VAS scores clustering between 88 and 89, leaning toward the “very happy” end of the scale. This stands in contrast to the previously published PEEEVS findings on visual field testing, which received a median VAS score of 67.5 and mean score of 60.45 (SD = 30.38) [1]. This difference aligns with existing literature, where visual field testing is ranked as one of the least preferred ophthalmic tests [5].

Older patients generally indicated more positive experiences with tonometry compared to younger patients. We speculate that this may be due to greater familiarity with the procedure from repeated testing over time, or directly due to reduced corneal sensitivity that can occur with increasing age [10]. Otherwise, no statistically significant differences in VAS scores were identified between different demographic categories for the remaining examination components.

The current study yielded a lower proportion of qualitative feedback across all questions, compared to the preceding paper on visual field testing. As a result, the thematic analysis was limited to the identification of only one prominent theme for most items. We speculate that the low rates of qualitative responses are a direct result of the higher VAS ratings provided by patients. Those who rated their experiences more favourably may have felt that their satisfaction was adequately conveyed through marking towards the ‘very happy’ end of the VAS, and additional commentary or elaboration may have been perceived as unnecessary. Conversely, for visual field testing in the preceding paper, patients may have been more inclined to provide detailed feedback as they perceived the open-ended prompts as an opportunity to voice concerns, frustrations, or desires for improvement. This pattern of response bias has been shown in the literature, describing an inverse relationship between a respondent’s rating of satisfaction and their likelihood of providing qualitative commentary [11].

Physical discomfort emerged as a recurring concern, particularly during slit lamp examination and OCT imaging. Specifically, the need to maintain a fixed posture during these tests can result in, or exacerbate, neck and back discomfort, echoing the challenges previously reported by patients undergoing visual field testing [1, 12, 13]. Therefore, it is important to pay particular attention to patients with pre-existing musculoskeletal conditions, ensuring that the setup is comfortable and allowing them to sit back and take breaks throughout the examination when needed. Practical adjustments may help mitigate some of these challenges, including the use of adjustable patient chair height, cushions for extra support and padding, and ensuring that patients’ lower extremities are supported with their feet firmly on the ground or footrest to enhance stability [14].

The slit lamp examination also posed unique challenges for individuals with certain body habitus, including those in late pregnancy. These patients describe having to lean forward in awkward or uncomfortable positions but tended to attribute these difficulties to their own physical characteristics rather than to the design limitations of the slit lamp. While not explicitly stated, this self-directed attribution may reflect underlying feelings of embarrassment or self-consciousness as they attempt to maintain the required head position for the examination.

Existing peer-reviewed literature on slit lamp ergonomics exclusively focuses on clinician experience, given the repetitive use and associated risk of musculoskeletal disorders [15]. However, the challenges reported by patients in this study highlight the need to also address patient-centred design constraints. Specifically, the need to develop more adaptable and ergonomic slit lamp configurations to accommodate individuals with different body profiles or pre-existing musculoskeletal conditions.

Regarding topical anaesthesia, patients reported transient stinging and burning sensations. While this is generally tolerable and consistent with known side effects of anaesthetic eye drops, individuals with sensitive or inflamed eyes described heightened discomfort. For these patients, the use of cooled preparations may be beneficial, as these have been shown to reduce stinging upon application [16, 17]. Furthermore, for patients who also experience stinging from mydriatic agents, instilling topical anaesthesia prior to mydriasis, particularly when both are required, may help to minimise the frequency and intensity of discomfort [18].

In regard to mydriasis, some patients reported feelings of disorientation and expressed concerns about safely navigating their environment. Eye patients with reduced visual acuity, and in particular those who have glaucoma, have been shown to experience heightened fear of falling and an increased risk of collisions [19**–**21**]**. Elderly individuals more broadly are also at greater risk of falling due to age-related declines in postural stability [20, 22]. This combination of factors highlights the need to ensure robust patient and caregiver education to mitigate the potentially heightened risks of accidents or falls following mydriasis.

As the PEEEVS was conducted in a private clinic setting, the participant population in this paper may overrepresent individuals from higher socioeconomic backgrounds, as well as those who have established long-standing rapport with their ophthalmologist. Additionally, the knowledge that one of the co-investigators was their treating clinician, even with assurances of anonymity, may have introduced bias towards more favourable ratings. In contrast, public healthcare settings typically involve clinicians with varying levels of training and experience, alongside systemic challenges such as resource limitations and higher patient volumes. These differences may significantly influence not only patients’ subjective experience of ophthalmic testing, but also their expectations regarding quality of care [23]. Further studies should therefore be adapted to include diverse population groups, particularly those in public healthcare settings and from varied socioeconomic and ethnic backgrounds. Comparing patient experiences between private and public sectors could provide valuable insights to identify areas for improvement and advocate for systemic changes where needed.

It is also important to acknowledge a potential selection bias in this study population. Individuals who have had extremely negative experiences with ophthalmic testing may have already disengaged with follow-up care, thereby excluding their perspectives from the study. Future research could address this by focusing on first-presentation patients or primary care ophthalmic settings, such as optometry practices, where a broader range of patient experiences, including those who may not arrive at specialised care, can be captured.

This study underscores the importance of evaluating patient experience during ophthalmic examinations, particularly in glaucoma care, where lifelong monitoring and adherence to testing and follow-up are crucial. While we have found that quantitative ratings for topical anaesthesia, OCT, tonometry, slit lamp examination, and mydriasis were generally favourable, qualitative analysis revealed persistent challenges for certain patient populations. Addressing these barriers will be key to improving the comfort, accessibility, and overall experience of ophthalmic testing for patients.

## Summary

### What was known before

- This is original research that addresses an important gap in the literature by exploring patient experiences of routine ophthalmic examinations.
- While these tests are essential to the diagnosis and management of eye disease, little is known about how patients experience them.

### What this study adds

- While participant experience of most tests were generally positive, certain components introduced distinct sources of discomfort for patients.
- This includes challenges with maintaining proper positioning during slit lamp examination and OCT, particularly for individuals with pre-existing neck or back issues, larger body habitus, or advanced pregnancy.
  - We outline practical recommendations that may improve patient comfort during routine eye examinations.

## Supplementary information

Supplementary table

## Data Availability

The datasets generated during and/or analysed during the current study are available from the corresponding author on reasonable request.
