# Supplementary material for: Patient experience of and barriers to the eye examination
Source: Eye (Lond). 2026 May 6;40(10):1552–8. doi: 10.1038/s41433-026-04499-w (PMC13342617; doi:10.1038/s41433-026-04499-w)

Supplementary information

Dunn's pairwise comparison for VAS score responses to question 8 (tonometry) between various age groups. Significant results (p < 0.05) are highlighted.


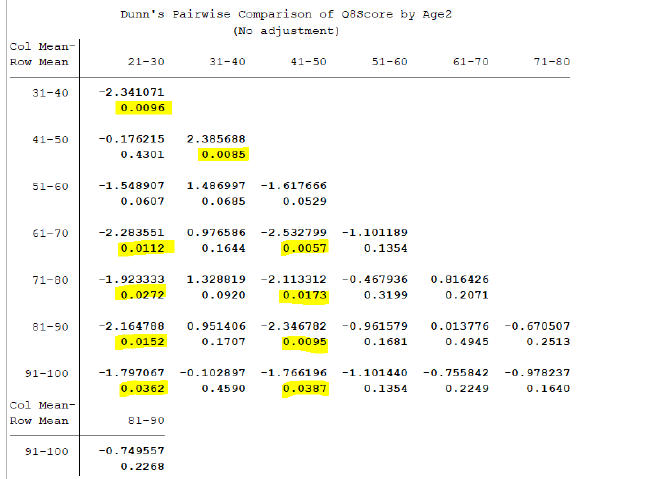

Supplement: Supplementary file 1 — Supplementary table [file 41433_2026_4499_MOESM1_ESM.docx]
